# First person – Siobhan Crilly

**DOI:** 10.1242/dmm.049479

**Published:** 2022-03-29

## Abstract

First Person is a series of interviews with the first authors of a selection of papers published in Disease Models & Mechanisms, helping early-career researchers promote themselves alongside their papers. Siobhan Crilly is first author on ‘ Zebrafish drug screening identifies candidate therapies for neuroprotection after spontaneous intracerebral haemorrhage’, published in DMM. Siobhan conducted the research described in this article while a postdoctoral research associate in Paul Kasher's lab at The University of Manchester, Manchester, UK. She is now an NC3Rs training fellow in the lab of Paul Kasher and Annalisa Tirella at The University of Manchester, investigating haemorrhagic stroke modelling.

**Figure:**
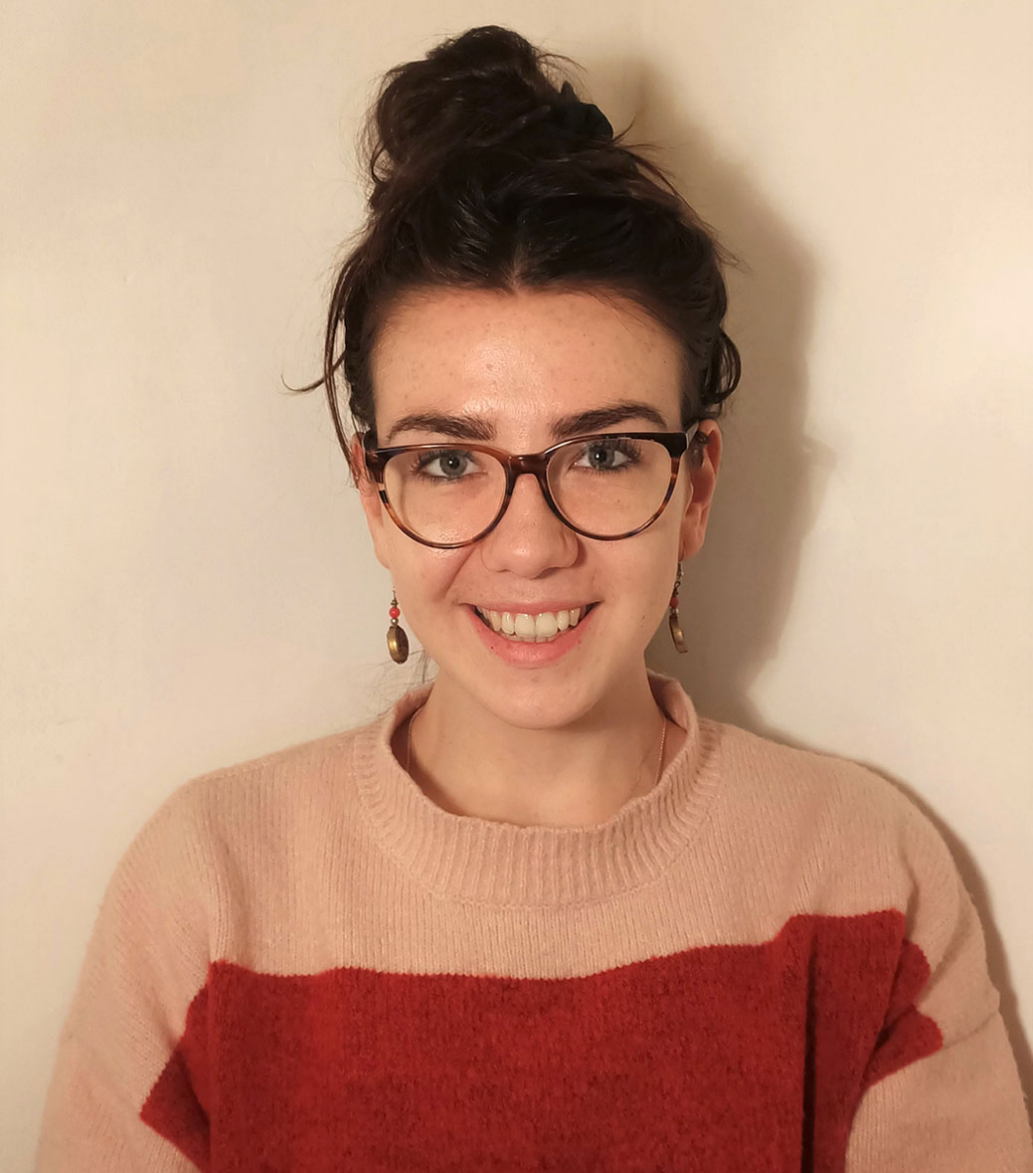
Siobhan Crilly

**How would you explain the main findings of your paper to non-scientific family and friends?**

People who suffer from a stroke have limited options for treatments, and so researchers are trying to find medications that can help prevent disability. We use a zebrafish to study stroke in the lab because they are see-through, so we can see the stroke and the brain while the animal is alive. In this paper, we used this model to screen 2000 medications that could potentially prevent disability after a stroke, which has never been done before. We found six drugs that were really good candidates and two of those drugs are often given to stroke patients to manage their blood pressure, so we analysed some data from a clinical trial to see whether the timing of giving these drugs was important for them to work. We saw that if patients are given these drugs after they have a stroke, similar to what we tried with the zebrafish, they are effective at preventing disability. This was excellent evidence to show that the zebrafish is a valuable model to investigate stroke.

“The drugs that have been identified in this screen may present new therapeutic approaches for stroke patients.”

**What are the potential implications of these results for your field of research?**

The drugs that have been identified in this screen may present new therapeutic approaches for stroke patients – some have never been used clinically before. We have also validated the zebrafish model as a valuable tool in the pre-clinical pipeline for stroke research.

**Figure:**
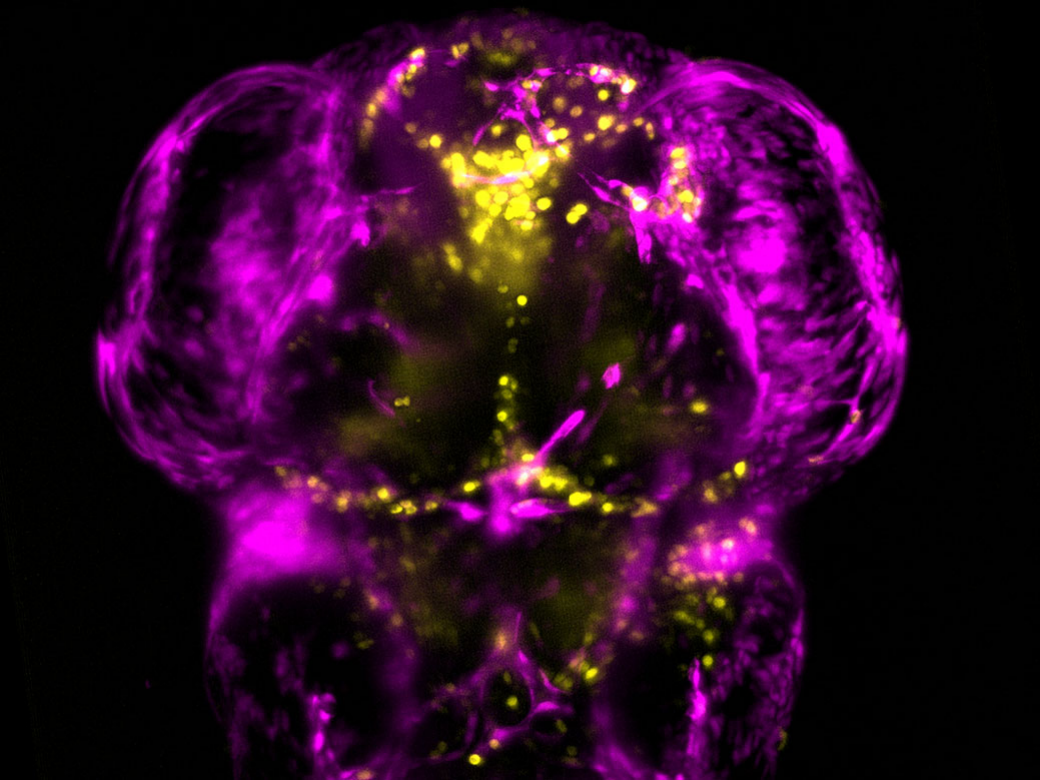
A bleed in the brain of a 3-day-old zebrafish.

**What are the main advantages and drawbacks of the model system you have used as it relates to the disease you are investigating?**

The zebrafish is a small aquatic vertebrate and shares 70% of genes with humans. We use the larvae, prior to 5 days of development, because they are such immature organisms and they are not considered a protected species. The larvae develop very quickly and have similar complex systems that we need to investigate stroke – a central nervous system, a circulatory system and an immune system. They offer many advantages over using rodents, and they are see-through, so we can image in a live animal and watch the progression of a stroke in real time. They are very easy to genetically manipulate, because the eggs are laid outside the female in large numbers. Also, zebrafish are innately regenerative and they recover fully from a stroke after 2 days, something humans find very difficult; this makes them ideal for finding new targets to help speed up healing.

**What has surprised you the most while conducting your research?**

What has surprised me most during this investigation is that from 2000 new and repurposable drugs, some of which have never been investigated functionally before, angiotensin-converting enzyme inhibitors (ACE-Is) were identified as potentially neuroprotective. ACE-Is are commonly prescribed for managing blood pressure, and, as high blood pressure is the main risk factor for stroke, a lot of patients are already taking ACE-Is when they arrive at hospital with a brain bleed. We have shown that ACE-Is are most effective as neuroprotective agents when given after a stroke, and this effect is lost if patients are already taking them beforehand. It is very important that we consider this timing effect in future pre-clinical and clinical trials.

**What's next for you?**

I now work on developing a model of brain bleeding that is entirely in a dish, using 3D modelling and biomaterials to recreate the damaged environment of a brain after a stroke. I hope that this will help advance stroke research further in the future, so that less animal experimentation will be necessary and drug discovery will be quicker.
